# Learning turning points—in life with long-term illness—visualized with the help of the life-world philosophy

**DOI:** 10.3402/qhw.v9.22842

**Published:** 2014-02-21

**Authors:** Mia M. U. Berglund

**Affiliations:** School of Life Sciences, University of Skövde, Skövde, Sweden

**Keywords:** Life-world philosophy, Gadamer, Heidegger, learning process, long-term illness

## Abstract

A long-term illness is an occurrence that changes one's life and generates a need to learn how to live with it. This article is based on an empirical study of interviews on people living with different long-term illnesses. The results have shown that the learning process is a complex phenomenon interwoven with life as a whole. The essential meaning of learning to live with long-term illness concerns a movement toward a change of understanding of access to the world. In this movement, in which everyday lives as well as relationships with oneself and others are affected, a continual renegotiation is needed. Texts from existential/lifeworld philosopher, Heidegger and Gadamer, have been used to get a greater understanding of the empirical results. These texts have been analysed with particular focus on learning turning points and the importance of reflection. The results are highlighted under the following themes: Pursuit of balance—the aim of learning, The tense grip—the resistance to learning, To live more really—the possibilities of the learning, Distancing—the how of the learning, and The tense of the learning—the whole of the learning. In those learning turning points are present. Knowledge from this study has been used to make a didactic model designed to give caregivers a tool to support patients’ learning. The didactic model is called: The challenge to take charge of life with a long-term illness.

A long-term illness changes one's life and generates a need to adapt. Patient experiences of living with long-term illness have been described in several studies (Thorne & Paterson, 2000; Thorne et al., 2002), but the learning process has not been stressed (Berglund & Källerwald, 2012). The learning process here is viewed as a complex phenomenon interwoven within every facet of life. Learning results in knowledge, understanding, the ability to think, and the ability to act (Berglund, 2011). Hörnsten, Sandström, and Lundman (2004) have described learning to live with a chronic disease as a development process, that is, in patients their illness interacts with their life in emotional and existential aspects. Hörnsten, Jutterström, Audulv, and Lundman (2011) describe that this interaction and self-management processes develop simultaneously. The interaction process is thereby important in learning to live with long-term illness. Till now, patient learning has not been the focus of attention, the consequence of which is that knowledge about patient learning has not formed a basis for the creation of educational programs and other forms of teaching materials in health services. Instead, patient education is often viewed from a biomedical perspective, or an *external perspective*, concerning illness and not on the contents of what patients really need to learn (Friberg & Scherman, 2005). In previous research, an imminent risk also emerged from patients being excluded from participating in their health care and treatment process (Berglund & Källerwald, 2012; Johansson, Dahlberg, & Ekebergh, 2006) wore seemed as not complied with the treatment. It is also a risk that the patients and the advice from the health care profession (Friberg & Scherman, 2005).

The focus of this article is to better understand the learning process concerning individuals suffering from a long-term illness. This starting point is based on a study in the thesis by Berglund (2011); this will be called the empirical study. This results of this study showed that the learning process was a complex phenomenon. The essential meaning behind learning to live with a long-term illness concerns movement toward a change in understanding the access to the world. In this movement, in which everyday lives and personal and intrapersonal relationships are affected, a continual renegotiation is needed. Learning includes developing an understanding of the relationship between the demands of life and those of the illness and is a response to the desire to live an ordinary life. To be able to move past the illness in favor of the patient's preillness life dictates a learning process where the person paradoxically allows the illness to take up more space in his/her life. Learning to balance a long-term illness with the rest of life is hard work and requires profound self-reflection. This self-reflection constitutes the instrument of learning, and the learning process can be both hampered and supported by other people. Learning entails that the individuals involved develop a new understanding of their self. A shift in paradigm of this magnitude can be difficult to define and study. Ekebergh (2001) maintained that most didactic studies do not reach the core of the complex problems related to learning; they research the “what” questions but not the “how” questions. Therefore, the aim of the present article is to describe what it means to learn to live with a long-term illness in its deepest sense.

1. Are there any significant learning turning points?
2. What role does reflection play in the learning process?

I will use this knowledge to develop a didactic model that will facilitate caregivers in supporting their patients while learning to live with a long-term illness.

## Scientific approach and method

The ontological and epistemological suppositions in this study are based on the life-world theory (Heidegger, 1927/1978; Husserl, 1907/1989; Merleau-Ponty, 1945/2002). In this theory, a person is seen as a *lived* body (Merleau-Ponty, 1945/2002). The lived body encompasses the physical, mental, and existential aspects of life at the same time. A person, according to Heidegger (1927/1978), is *thrown in* to the world and *thrown out* from his/her actual situation. A person's life can, thus, be said to be determined and undetermined. A person's life gets its meaning from the knowledge that life is spiritual. A person lives his/her life as an active agent together with other people. A person is a provider and a seeker of knowledge when he/she wants to take care of, comprehend, and understand life. When a person suffers from a long-term illness, the body is changed and, thus, their access to and interaction with the world is changed as well. The experiences that people have when they are living with a long-term illness are seen, in this research, to constitute a possibility for learning. In the life-world, people take their natural attitude toward the world of things for granted (Bengtsson, 2006). The theory of intentionality helps to understand how people experience the world. According to this theory, man is directed toward something other than itself, and the natural setting is to not reflect on everything. Human beings learn through their experiences and incorporate these experiences as a habit. Humans can also learn in a more reflective manner. The phenomenological attitude requires the researcher to emerge from the natural setting and adopt a reflective stance. This is done by questioned and problematized the understanding process (Dahlberg & Dahlberg, 2003).

The life-world theory also guides the approach to learning expressed in this study. The learning process is seen as a complex phenomenon that involves the whole being of a person. Learning is an embodied process of understanding that includes thoughts, experiences, and feelings. Learning entails a change, which occurs with the help of reflection and dialog (Bengtsson, 2006; Ekebergh, 2007; Kroksmark, 2007). This means that how people learn depends on the individual's experience and understanding of what is to be learned. What and how a person learns differs with individuals, even if learning often takes place in relation to other people.

In this study, the Reflective Life-world Research (RLR) (Dahlberg, Drew, & Nyström, 2008) is used. RLR is based on a phenomenological philosophy with a life-world theoretical foundation. The overall aim of RLR is to describe and clarify lived experiences in such a way that the knowledge of a person's existence and experiences is increased. The reflective life-world approach is characterized by a search for meaning and a focus on the phenomenon. The phenomenon's essential meaning is to be understood as the actual phenomenon in relation to its specific contexts. Dahlberg (2006) maintained that the research activity was based on the concept that meaning belongs to the life-world and exists in the relationship between the subject and the phenomenon, which, in this context, represents the researcher and the phenomenon. To be able to see and understand the meaning of the phenomenon, it is necessary to be open for what emerges.

### Method to elucidate the empirical results using the life-world philosophy

The method can be characterized as a philosophical elucidation. This study takes its starting point from the result of an empirical study about humans’ experiences of living with a long-term illness (Berglund, 2011). In the empirical studies, interviews were performed. Nine persons who were interviewed were between 21 and 84 years old: there were five men and four women. They had different long-term illnesses, such as rheumatoid arthritis, Bechterew disease, chronic obstructive pulmonary disease, multiple sclerosis, and diabetes. The data were analysed with regards to the essence of the phenomenon and its meaning elements. The intention was to describe the lived experience as thoroughly as possible. In the results from this empirical study, learning emerged as being tightly interwoven with life in general and affected the sufferers deeply.

To be able to gain a greater understanding, the results of the empirical studies here have been analysed in terms of texts from existential philosophers. Text from philosophers has been used to deepen the understanding of the empirical data (Adolfsson, 2010; Karlsson, Ekebergh, Larsson Mauléon, & Almerud Österberg, 2012) or to get a deeper understanding of phenomena of caring (Sarvimäki, 2006). To gain a greater understanding of what learning to live with a long-term illness means, texts from existential philosophers were used including Heidegger's (1927/1978) *Being and Time* and Gadamer's *Truth and Method* (1997) and *The Enigma of Health* (2003). Heidegger and Gadamer are existential philosophers who clearly have roots in the life-world theory. This study used these philosophical works to make associations with the empirical results of the study by Berglund (2011). Two main questions that emerged from the research are the aims of this study: 1) Are there any significant learning turning points? 2) What role does reflection play in the learning process? These questions were useful in focusing both the empirical results and the philosophical works to gain a deeper understanding of the learning process. This analysis of this paper is in accordance with the life-world approach (Dahlberg, 2006; Dahlberg et al., 2008). Overall, the analysis can be described as a movement between the empirical results and the philosophical works. A bridled approach has been used throughout this process to maintain openness and to not make definite what is indefinite too quickly (Dahlberg & Dahlberg, 2003). For example, is this a learning turning point? What makes it as it is? Can it be understood in another way? A focal point in the analysis is that each part is understood in relation to the whole, and the whole is understood in relation to its parts during the process; in this way, one's understanding of the whole will be changed (Dahlberg et al., 2008). The results are presented as six themes and include quotations from the empirical study to deepen the magnitude of the results of this study. The themes are created to describe the learning process and what is involved, for example, *Pursuit of balance: the aim of learning*. Each theme concludes with a number of points that summarize the learning turning points that emerged in the philosophical elucidate.

The ethical considerations for the empirical study, including the requirements for information, consent, confidentiality, and use, are based on the ethical principles of research of the Swedish Research Council (2002) and the Helsinki Declaration (World Medical Association, 2000). Informed consent was received from the informants that participated in the empirical study (Berglund, 2011) both orally and in writing. Further ethical considerations concerned accuracy in relating the empirical results and not misrepresenting or overinterpreting their meanings when associating them with the philosophical texts.

## Results

The results are presented as five themes:

- Pursuit of balance: the aim of learning
- The tense grip: the resistance to learning
- To live more authentic: the possibilities of learning
- Distancing and reflecting: the how of learning
- The tense of learning: the whole of learning

### Pursuit of balance: the aim of learning

A diagnosis of long-term disease can feel disruptive to a person who takes health for granted. This disruption is recurring because of the nature of long-term illnesses. Patients must repeatedly learn to handle new symptoms and problems caused by their disease. Learning in form of changed understanding and ability to deal with the new situation seems similar to how Gadamer (2003) described health. He believed that health is reflected in well-being, when the person is being creative, curious, and, thus, forgetting about himself/herself. The empirical results showed that a person believed that he/she had learned to live with the disease when they no longer needed to think about it or the problems it causes in their lives. This learning becomes possible when the knowledge associated with the disease and the coping strategies become inherent in the body. Gadamer's (2003) description of health makes it possible to understand why learning is intertwined with life in general. He further described health as a state of balance in terms of wholeness and belonging to a meaningful context. For a person with a chronic disease, a disruption in the aforementioned balance may occur when a new disease is discovered or when his/her symptoms become complicated. To regain a sense of balance, the patients must learn to deal with the changed situation and try to find a new balance in their life.

To get a balance in life, a person needs to learn how to detect and evaluate what is important for his/her well-being. One woman in the empirical study described how she tried to maintain balance in life by considering her needs more and creating more room in her life. This was important for her to feel good about herself, provide strength to her family, and decrease the attention on the disease. By learning to prioritize differently, she acquired a change in understanding what was important in life. This altered understanding is accomplished through reflections on inner dialog, in dialogs with other people and with the support of diary writing.

To maintain a balance in life, it is also important to appreciate what was previously held as important in life. One man in the empirical study explained how he balanced and revaluated his life to cope with living with long-term illness. He illustrated that as a “negative account” with disease and discomfort and a “positive account” with the positives in life. Then, the value of the negative account was loaded on the thinks he had on the positive account was loaded up. This can be understood as the maintenance of balance. When asked if he usually thought in this way, the subject said it was only now, when he was talking about it, that it became clearer. Here emerges the importance of reflection, dialog, and socializing with others for learning.

Being active is an important aspect of good health. To be active is as Heidegger (1927/1978) means “a natural way to be a human”. Heidegger describes humans as an agent in the world who acts and uses things (don) as tools in a context that makes them meaningful. This manifests itself as a desire to hold on to their work and continues to be active in their life. With the help of Gadamer's (2003) descriptions of health, exercise and the ability to work can be understood as a person's opportunity to be themselves and as a way to maintain balance in life. But the importance of being active can also be a burden, for example, when the person does not balance the importance of being active and the importance of resting. By reflecting on this, the importance of balance becomes a learning experience.

Caring for themselves and others is a part of performance of activities. The empirical results have shown that people often consider caring for themselves as a second priority to caring for others, things, and activities. The texts of Heidegger (1927/1978) express this behavior as a way to escape the anxiety and responsibility of taking care of one's self. This has also been described as removing the focus from the disease so that it does not take too much space later in life. Too much space is accorded only if the illness gets worse. In this reflection, there is a balance emerging that is important for future health.

To obtain insight into the way the person with an illness prioritizes and evaluates what is important for their own well-being, dialog and questions emerge that are important. Questions that cause reflection have an important place in learning. An example of a challenging question is, “Why would you not be able to work as a midwife?” This question starts a reflection where the pros and cons are weighed against each other. The outcome of this case was that a woman with multiple sclerosis who wanted to continue her education commenced with a new profession. Reflection on their own opportunities gives people with long-term illnesses the courage and strength to make decisions and challenge themselves to find balance in life.

- In the quest for balance, insights about what is important in life emerge as learning turning points. These are insights that can help the learner to prioritize in a new way.
- A learning turning point is when words are put to experiences and are visualized.
- A learning turning point occurs when a person becomes aware of the importance of balancing the performance motivation against the risk of deteriorating health.
- A learning turning point is when a person becomes aware of how he/she prioritizes his/her own care in their lives.
- A learning turning point can be questions that challenge people's understanding of their lives.

### The tense grip: the resistance to learning

In life, there are unconscious goals for all humans: to be well, to be developing, and to be growing as a person. These goals equate to being in good health. According to Gadamer (2003), disease opposition refers to something that intrudes in life and something that the person does not want to reconcile with in his/her life. The results of the current study reveal a tension in individuals with chronically illness between the goal of health and the wish to retain their preillness life; this tension signifies that the person does not want to change and evolve because it is the disease that dictates that change. From this perspective, an embedded resistance to learning is easier to understand.

A man who has had diabetes since he was a child can exemplify this. In an unreflective way, he let the familiar life take precedence while the disease perpetuated. He felt it was important to “be like everyone else.” After he became mature, he was able to see that ignoring the illness caused several organs to fail. He expressed that he “realized too late” the importance of taking care of himself and his illness. He argued that it was only when the disease threatened his life that he was able to make other choices. If he had made different choices, the disease may not have developed as rapidly or in the same way that it did. In this case, the learning experience was “hard-earned.”

How can the desire to maintain the preillness life be understood? According to Gadamer (2003), humans live in a manner that allows them to feel at home. When a disease enters a person's life, the person can feel homeless. Life and health, if not reflected upon and taken for granted, become extremely important and what they now strive to preserve. Disease, according to Gadamer (2003), can be described as the loss of an undisturbed perception of living life freely. The results showed how people are pressed to make decisions and learn how to handle problems in their everyday lives concerning obstacles they were not required to consider previously. The unwillingness to give up their preillness life may be rooted in not wanting to recognize the presence of the disease, weakness, or a need for assistance. For resistance and vulnerability to be recognized, these aspects of a patient's inability to move on need to be made visible to allow for reflection. Resistance to diseases, to learning, and to change is rarely discussed in patient education. Patients are assumed to have accepted their disease and, thus, are willing to learn about it. The empirical result shows how patients seem to “listen and learn” about their disease, but in actuality, the information works to distance them from the disease all the more. Teaching gives opposite effect than what it intends to provide.

One man in the empirical study talked about his fears of health care and disease, but these fears never attracted the attention of health care professionals. Instead of asking for help, he hurried home and consumed alcohol or exercised excessively. For him, it took a long time to reach a learning turning point. His fear of knowledge about the disease manifested into an unwillingness to change and risk of no longer recognizing himself. This fear may be better understood with the help of Gadamer's (2003) homeless analogy regarding disease diagnosis.

According to the empirical results, people often imagine a dark and scary picture of their future with the disease. This image is often unwavering and is rarely discussed. The person does not want to ask about the future because they are afraid their imagination will be confirmed, and they want to continue living under the guise that this condition is not happening to them. However, the results showed that talking about their concerns for the future and gaining knowledge about other possible future scenarios have important implications for patients regarding learning and the ability to deal with their fears. Discovering a different view of the future helps them accept their fate more easily.

- The results showed that it was only when a person felt his/her life was threatened that it becomes easier to prioritize his/her health and life and, thus, create space for themselves and their well-being. Thus, the realization that his/her existence is threatened is a learning turning point.
- A learning turning point that emerges is the recognition of the disease and their own vulnerability.
- A turning point is also learning to confront their fear of change. In the confrontation also the convulsive go of the pre illness life made visible and a new understanding of the current situation reached.
- A significant turning point in learning is when the dark picture of the future changes to a more varied picture.

### To live more authentic: the possibilities of learning

Despite the fact that the well-known, preillness life is important, disease can be seen as an opportunity to develop and live a life more authentic. According to Heidegger (1927/1978), people are thrown into the world and forced to live life under certain cultural and historical traditions. People cannot escape their factuality, but they can face it in two ways. People can live life without thinking about their factuality, which is what they mostly do. Heidegger calls this “Das Man,” which means living inauthentic. People can also strive to be themselves and act responsibly, which he called living in an authentic existence. The empirical results showed that the illness pushes the realization of one's own facticity and its terms forward. The disease is also pushing the person to live in a more authentic manner. A significant aspect of learning is a patient's awareness of opportunities and possibilities to influence and control their lives and goals.

A person with long-term illness is forced to understand the conditions for his/her own existence. That is to say that life is finite and that there are opportunities to choose how life should be lived. An example of this is a woman who said that because of her disease, she was forced to stay up and learn more about herself: “...If I had not stayed up, I had not known what I know today...” Today, she is making different decisions prioritizing the different aspects of her life to create more time for herself so that she can continue to feel good. She described feeling safer and more sure of herself. Other lessons that emerge are a greater understanding of other people's lives and actions, as well as the need to take advantage of the time left and live more in the present. Thus, learning for those with long-term illnesses can be seen as an opportunity to reflect on their way of life, become aware of their existence, and develop as a person.

The unwillingness to learn about the disease and recognize that it exists can be expressed as an escape from the responsibility of their own existence, according to Heidegger (1927/1978), but this escape is not possible. Heidegger described this escape as living an unauthentic life or living as others live. When time is regarded as never ending, as healthy individuals regard it, the thought of death is far from the mind. The question becomes whether a diagnosis of long-term disease, by its nature, for a time allow the person to live as other but than the disease get worse or than life are threatened the person have live more authentic as “I myself.”

Attempting to live, as others do, can be understood as the difficulty and reluctance to make decisions that differ from others. According to Heidegger's reasoning, people are aware of their factuality and, therefore, the ability to live more suitably may come from using the word “I” instead of others.

When people become more aware of themselves, they seem to make more informed choices and live more real existences. Reflection helps a person to become aware of his/her decisions. The more conscious the decision, the safer it is for the person and for others. According to Heidegger, people live in a movement between this two way to be. Heidegger does not assess the different ways to be, but in a life with long-term illness, the importance of living a more authentic life is paramount for an individual's health condition and life, in general.

The question is what direction life will take. In results, goals related to being able to manage something that the person wants to do are important for the learning process. Often, the goal is to retain independence. However, a change in understanding, that is, to have reached self-awareness and developed as a person, does not emerge as a goal for learning in life with illness. This goal is often made explicit in other learning contexts and can be visual in learning to live with long-term illness. By creating learning goals, patient education can be supported. With clear goals, it becomes possible for a person to make decisions and live more real.

- A learning turning point is the realization of one's own factuality and the ability to live more real.
- A learning turning point is when the person becomes aware of the “I myself” and not “others,” in general.
- A learning turning point is when a person becomes aware of the importance of his/her decisions. Illness can thus be seen as a help to take the helm and steer their lives.
- A learning turning point is the consciousness of learning objectives.

### Distancing and reflecting: the *how* of learning

To understand the *human being-in-world*, Heidegger (1927/1978) described how things are used in an unreflective way. It is through this use that they get their meaning. He called this *ready-to-hand*. When one thing breaks, people begin to examine it and see it more as an object and reflect on it. Heidegger called this *present-at-hand*. The goal is often focused on restoring the function of the thing and, thereby, reverting it to a ready-to-hand perspective. The results from the empirical study show that a body part or function of the body becomes visible only when it no longer functions normally. It takes time away from a quiet working state and becomes noticed. The person tries to acquire knowledge to regain control and to be able to use the “broken part” in an unreflective way to feel whole again. An example of this, taken from the results, is pain in the hip. The person who is in pain has to constantly think about how they walk, such as which leg to start with on the stairs. When the pain is gone, this sensation is incorporated in the body, returning the leg to a ready-to-hand state, and the person no longer needs to think about how they walk down the stairs.

Diseases show up in different ways, but persistent diseases usually deteriorate with time and create new problems in life. In connection with impairments, commuting the person between to distance the “broken” part of the body, the disease or the obstacles of everyday life caused by the disease in a present-at-hand way and to an unreflective present in a ready-to-hand way. This present-at-hand and ready-to-hand can be seen as a way to describe the learn process of how to manage illness and the changing body. It can also be described as a movement between feeling whole and at home and feeling fragmented and homeless, as Gadamer (2003) expressed. Distancing of the body and its functions gives the person the opportunity to reflect on them, which is important for the learning process.

The person distancing and objectifying the disease creates opportunities to reflect and put together different aspects for a new understanding concerning themselves. In this way, the disease can be visualized and studied to be integrated into a new whole. The new whole is created with the help of distancing, which gives an altered perception that is important for the learning process. However, for this to be recognized as learning requires that it is articulated.

- A learning turning point is seen in the distant way a patient relates to the illness or part of the body.
- A learning turning point is when the patient becomes aware of his/her new whole and changes his/her understanding of being-in-world.

### The tense of learning: the whole of learning

Existence is characterized, according to Heidegger (1927/1978), as time and its phases, or existence tenses: past tense, present tense, and future tense. These phases are intertwined and form a unit. The past is something that has to be processed again. If there were no past, human life would be devoid of context, according to Heidegger. Education and information given in health care are mainly focused on the present, where the disease is explained from a biological perspective. The information given is also directed to the future, forecasting information about what patients can do to avoid deterioration. Remarkably, the patient's fear and thoughts of the future are rarely affected. The same applies to patient's previous experiences. An important aspect of time (past tense) is not included in teaching situations. An understanding of integrating new experiences with patients’ previous experiences, that is, their traditions, seems to be lacking. According to Gadamer (1997), development lies in the meeting of the former experiences with the new experiences. Caregivers’ understanding of and attitude toward existence tenses may, thus, become a problem for the patient's ability to learn. Because parts of existence tenses are lost in teaching situations, further fragmentation and feelings of homelessness are likely.

Health professionals need to start asking for patients’ past experiences, how they are dealing with their illness, and what they think about their future. The experiences must be seen as relating to human existence, thoughts, feelings, and actions in all tenses. To support patients’ learning, past experiences need to be reflected on, studied, and integrated with the new experiences and thoughts about the future. Then, a holistic approach based on the conditions of existence can be considered.

- Learning turning points in conversations involving patients’ thoughts and feelings about the past, present, and future are important.

## Discussion

The life-world philosophical elucidation has shown that reflection plays a decisive role in learning to live with a long-term illness. Thoughts, feelings, and actions become more conscious by reflecting on experiences. The one who is learning encounters the resistance that the illness constitutes in the reflection. The resistance can be seen as a way to help stop, reflect, and learn about oneself. It is made clear in the reflection that it is the person that must choose and make decisions in his/her life. The learning process requires an active effort to make the disease visible in the person's life. The decisive factor for initiating and supporting the advent of reflection is challenging questions. These are questions that they get from others or simply ask themselves. The challenging questions confront the learner with the actual situation and the impossibility of not making changes to achieve well-being. The reflection and dialog has an important role in learning to live with long-term illness, this role has earlier been stressed as important in students’ learning process (Bengtsson, 2006; Ekebergh, 2007; Kroksmark, 2007).

The results have shown that learning emerges from learning turning points. Especially important turning point is when the sufferer reaches the realization that it is impossible not to change, the illness is there and it will affect them and their life regardless of what they want. It affects the situations, possibilities, and responsibilities for making choices. A characteristic of learning turning points is that they challenge a person's understanding, make demands on their responsibility, and confronts the learner's thoughts and feelings. The learning demands that the person reflect deeply about his/her life, spirituality, and responsibility to make decisions. To live an untroubled life becomes more difficult with the insight about how choices affect health. Learning, however, also provides the possibility for assessing life in a different way and for providing time for what the person considers to be significant in life. Learning, thus, provides the opportunity to take charge of one's life. The opportunity for a more real life and to achieve well-being in life with long-term illness becomes possible. Jutterström, Isaksson, Sandström, and Hörnsten (2012) have described turning points in self-management of type 2 diabetes as a sudden occurrence of a need to act and take responsibility. They mean that a turning point was when the patients integrated the illness emotionally, existentially, and in practice. This has similarities with the present results. In which learning tourning points sometimes appear suddenly, sometimes they is maturing. The learning turning points are several and they are all important for the learning process and the willingness to take responsibility for one's own health.

The results have mainly dealt with genuine learning, which I maintain is something that differs from the learning of information. The information society of today makes it relatively easy for patients and relatives to seek and get hold of information all by themselves about illness and treatment. There is nothing that emerges in the results that would indicate a lack of information. Previous research (Johansson et al., 2006; Skalla, Bakitas, Furstenberg, & Ahles, 2004) has shown that patients do not lack information about illness and treatment but that they still have problems in translating the information to their own lives. From the results, it can be seen that information and teaching are directed toward what Eriksson (1989/2000) called as the “doing” level. A level where patients are expected to do what they are told to do, which is not the same as genuine learning. The genuine learning exists on what Eriksson (1989/2000) called the “being and caring” level. This has been seen in the results to be a learning that deeply challenges and affects the patient, a learning that makes the guilt and responsibility visible as part of a person's existence. The genuine learning has emerged in the results when the sufferers have gone from having ignored their situation as being ill to living more really.

From a lifeworld perspective in caring science, I would maintain that learning should be seen as a process where the individual gains competency in coping with his/her life with long-term illness. Based on the results, it is a long process where the need for knowledge and understanding in relation to the illness constantly changes. Marking the starting point as emanating from the patient's life context and understanding of his/her situation can support the learning. This means that the carers should proceed from the patient's need for learning and not from readymade programs. In the learning process, the whole individual, that is, his/her experiences of and thoughts about the past, present, and future have to be involved. The life conditions and the possibilities have to be challenged and reflected on in order for it to be a genuine learning. An explicit caring science, lifeworld didactic perspective, is needed in patient education. Dahlberg, Todres, and Galvin (2009) argued for a lifeworld-led care where the focus is on health instead of illness and where the philosophy of the person is seen and the care is consistent with this, that is, the learning support also has to be consistent with this, if we want to achieve genuine learning.

There are possibilities of supporting a person's learning when suffering from a long-term illness, with a didactic approach, which is based on lifeworld theory and which has an ethical patient perspective. In order to be able to support learning at the existential level, a conscious didactic approach is needed. This didactic approach is formed by tactfulness and challenges and presupposes a long-term caring relationship.

A tactful approach has previously been described by van Manen (1993) and Ekebergh (2007). This means being able to sensitively understand the learner's world and his/her way of learning and understanding. In a tactful approach, the attention is directed toward the patient as a lived body. The encounter is characterized by openness and sensitivity for how the patient speaks about himself/herself and his/her illnesses/difficulties. Attention is directed toward how the patient speaks of himself/herself as *one* or about his/her illnesses/difficulties as an *it*. The tactfulness gradually develops during the encounter and does not have any set rules but presupposes that the carer consciously reflects in order to be open for the patient's understanding of his/her situation and thoughts of the future. The patients’ facticity and possibilities can be observed with a supportive but at the same time challenging approach. It is in the dialog that the reflection is initiated, often with the help of questions. Questions that support the reflection's movement toward a greater understanding are challenging in a considerate way. In the dialog, the understanding of the lived body reaches deeper levels, such as illness, impediments, behavior, thoughts, and feelings. In the dialog the patient is challenged to make decisions and formulate his/her own aims. The task of the carer is also to help the patient to see his/her own learning and development. The confirming approach is an important tool in doing this. According to van Manen (1993), the confirmation of the other in the tactful approach is based on perception and sensitivity.

A tactful, challenging approach also entails waiting. According to Bullington (1999), the ability to listen to the lived body is the most important tool in psychosomatic treatment. This means that the carer has patience and waits for the patient. However, I maintain that the carer can, via questions that initiate reflection, support the patient at an early stage to listen to the lived body. Bullington (1999) states that carers have to endure not being able to fix the patient's objective body and discontent. The carer should instead believe in the process and in the patient's ability to create meaning. I maintain that this is important but that the patient should not be left alone in this process, as has been shown in the results in the present thesis. In order for the carer to be able to have a tactful and challenging didactic approach, where patients’ understanding is challenged on an existential level, it is necessary that the carers learn to support it.

## Conclusion—a didactic model: the challenge to take charge of life with a long-term illness

The philosophical concepts and relations to learning turning points, along with knowledge about caring, didactics, and the life-world theory, formed the basis of didactic model of this study. The model consists of four theses that can also be viewed as the conclusion of this study.

First thesis: Confronting one's life situation and challenging to make a changeThe didactics make the facticity and challenge the impossibility of not making changes

- Confronting the facticity
- Discerning and challenging fear
- The role of the questions

Second thesis: Positioning oneself at a distance when creating a new whole

The didactics support a distancing, where resistance can be made aware of and studied in order to create a new whole.

- Distancing
- Present-at-hand and ready-to-hand
- The movement of the reflection

Third thesis: Developing self-consciousness and taking responsibility

The aim of the didactics is to clarify the patient's own responsibility and to support a reflective approach where the person goes from saying “one” to saying “I,” which constitutes learning at an existential level.

- Noticing how the patient talks about himself/herself and the illness
- Clarifying one's own responsibility
- Expressing in words

Fourth thesis: Making learning visible with the aim of providing development and balance in life.

The aim of the didactics is to make learning visible with the aim of providing a possibility to feel that one is developing and has balance in life.

- Noticing learning as change and development
- Setting goals and evaluating

Learning to live with long-term illness creates possibilities. The knowledge and experiences that confrontation with facticity results in constitute insights on life's spirituality and on what is valuable. The learning thus contributes to the possibilities for changes and new priorities in life. The learning supports a greater understanding of oneself and of others. The learning results in the possibility of taking charge and of steering one's life toward new goals. The didactic model must be developed and can then be a tool for caregivers in their efforts to support patient's learning with the aim to achieve optimum wellness. The new perspective on learning to live with long-term illness, presented in the model “The challenge: to take charge of life with a long-term illness,” puts demands on the care organization and a caring that is based on the patient's needs and not only on the diagnosis of the illness.

## References

[CIT0001] Adolfsson A (2010). Applying Heidegger's interpretive phenomenology to women's miscarriage experience. Psychology Research and Behavior Management.

[CIT0002] Bengtsson J (2006). The many identities of pedagogics as a challenge: Towards an ontology of pedagogical research as pedagogical practice. Educational Philosophy and Theory.

[CIT0003] Berglund M (2011). Att ta rodret i sitt liv—lärande utmaningar vid långvarig sjukdom [*Tacking charge of one's life*—*challenges for learning in long*—*term illness*].

[CIT0004] Berglund M, Källerwald S (2012). The movement to a new understanding: A life-world based study about how people learn to live with long-term illness. Journal of Nursing Care.

[CIT0005] Bullington J (1999). The mysterious life of the body: A new look at psychosomatics.

[CIT0006] Dahlberg H, Dahlberg K (2003). To not make definite what is indefinite: A phenomenological consequences in human science research. The Humanistic Psychologist.

[CIT0007] Dahlberg K (2006). The essence of essences: The search for meaning structures in phenomenological analysis of life-world phenomena. International Journal of Qualitative Studies on Health and Well-Being.

[CIT0008] Dahlberg K, Dahlberg H, Nyström M (2008). Reflective life-world research.

[CIT0009] Dahlberg K, Todres L, Galvin K (2009). Lifeworld-led healthcare is more than patient-led care: An existential view of well-being. Medicine. Healthcare & Philosophy.

[CIT0010] Ekebergh M (2001). Tillägnandet av vårdvetenskaplig kunskap: reflexionens betydelse för lärandet.

[CIT0011] Ekebergh M (2007). Lifeworld-based reflection and learning: A contribution to the reflective practice in nursing and nursing education. Reflective Practice.

[CIT0012] Eriksson K (2000). Hälsans idé.

[CIT0013] Friberg F, Scherman M. H (2005). Can a teaching and learning perspective deepen understanding of concept of compliance? A theoretical discussion. Scandinavian Journal of Caring Sciences.

[CIT0014] Gadamer H.-G (1997). *Sanning och metod* [Wahrheit und Methode] [Truth and method] (A. Melberg, Trans.).

[CIT0015] Gadamer H.-G (2003). *Den gåtfulla hälsan: essäer och föredrag*. [Über die Verborgenheit der Gesundheit] [The Enigma of Health] (J. Jakobsson, Trans.).

[CIT0016] Heidegger M (1978). Sein under Zeit [Being and time: Part 1 and 2].

[CIT0017] Hörnsten Å, Jutterström L, Audulv Å, Lundman B (2011). A model of integration of illness and self-management in type 2 diabetes. Journal of Nursing and Healthcare of Chronic Illness.

[CIT0018] Hörnsten Å, Sandström H, Lundman B (2004). Personal understanding of the illness among people with type 2 diabetes in Sweden. Journal of Advanced Nursing.

[CIT0019] Husserl E (1907). Fenomenologins idé [Die idee der phänomenologie] (J. Bentsson, Trans.).

[CIT0020] Johansson A, Dahlberg K, Ekebergh M (2006). The meaning of well-being and participation in the process of health and care women's experiences following a myocardial infarction. International Journal of Qualitative Studies on Health and Well-Being.

[CIT0021] Jutterström L, Isaksson U, Sandström H, Hörnsten Å (2012). Turning points in self-management of type 2 diabetes. European Diabetes Nursing.

[CIT0022] Karlsson A.-C, Ekebergh M, Larsson Mauléon A, Almerud Österberg S (2012). Only a whisper away. A philosophical view of the awake patient's situation during regional anaesthetics and surgery. Nursing Philosophy.

[CIT0023] Kroksmark T (2007). Fenomenografisk didaktik—en didaktisk möjlighet [Phenomenographical didactics - a didactic ability]. Didaktisk Tidskrift.

[CIT0024] Merleau-Ponty M (2002). Phénoménologie de la Perception.

[CIT0025] Sarvimäki A (2006). Well-being as being well—a Heideggerian look at well-being. International Journal of Qualitative Studies on Health and Well-Being.

[CIT0026] Skalla K. A, Bakitas M, Furstenberg C. T, Ahles T (2004). Patients’ need for information about cancer therapy. Oncology Nursing Forum.

[CIT0027] The Swedish Research Council (2002). Forskningsetiskaprinciper inom humanistiska-samhällsvetenskaplig forskning. http://www.codex.vr.se/texts/HSFR:pdf.

[CIT0028] Thorne S, Paterson B, Acorn S, Canam C, Joachim G, Jillings C (2002). Chronic illness experience: Insights from a qualitative meta-study. Qualitative Health Research.

[CIT0029] Thorne S. E, Paterson B. L (2000). Two decades of insider research: What we know and don't know about chronic illness experience. Annual Review of Nursing Research.

[CIT0030] van Manen M (1993). The tact of teaching. The meaning of pedacogical thoughtfulness.

[CIT0031] World Medical Association (2000). Declaration of Helsinki. http://www.wma.net/en/20activities/10ethics/10helsinki/index.html.

